# Direct Detection of 4-Dimensions of SARS-CoV-2: Infection (vRNA), Infectivity (Antigen), Binding Antibody, and Functional Neutralizing Antibody in Saliva

**DOI:** 10.21203/rs.3.rs-3745787/v1

**Published:** 2023-12-28

**Authors:** Aida Mohammadi, Samantha Chiang, Feng Li, Fang Wei, Chang S. Lau, Mohammad Aziz, Francisco J. Ibarrondo, Jennifer A. Fulcher, Otto O. Yang, David Chia, Yong Kim, David T.W. Wong

**Affiliations:** 1 School of Dentistry, University of California Los Angeles, Los Angeles, CA, USA; 2 GenScript USA Inc.; 3 Division of Infectious Diseases, Department of Medicine, David Geffen School of Medicine, University of California Los Angeles, Los Angeles, CA, USA

## Abstract

We developed a 4-parameter clinical assay using Electric Field Induced Release and Measurement (EFIRM) technology to simultaneously assess SARS-CoV-2 RNA (vRNA), nucleocapsid antigen, host binding (BAb) and neutralizing antibody (NAb) levels from a drop of saliva with performance that equals or surpasses current EUA-approved tests. The vRNA and antigen assays achieved lower limit of detection (LOD) of 100 copies/reaction and 3.5 TCID₅₀/mL, respectively. The vRNA assay differentiated between acutely infected (n=10) and infection-naïve patients (n=33) with an AUC of 0.9818, sensitivity of 90%, and specificity of 100%. The antigen assay similarly differentiated these patient populations with an AUC of 1.000. The BAb assay detected BAbs with an LOD of 39 pg/mL and distinguished acutely infected (n=35), vaccinated with prior infection (n=13), and vaccinated infection-naïve patients (n=13) from control (n=81) with AUC of 0.9481, 1.000, and 0.9962, respectively. The NAb assay detected NAbs with an LOD of 31.6 Unit/mL and differentiated between COVID-19 recovered or vaccinated patients (n=31) and pre-pandemic controls (n=60) with an AUC 0.923, sensitivity of 87.10%, and specificity of 86.67%. Our multiparameter assay represents a significant technological advancement to simultaneously address SARS-CoV-2 infection and immunity, and it lays the foundation for tackling potential future pandemics.

## Introduction

The significance of affordable diagnostic tools capable of identifying SARS-CoV-2 RNA, antigen, and host-generated antibodies has been highlighted by the COVID-19 pandemic. The clinical progression of SARS-CoV-2 infection involves an initial phase with detectable viral RNA (vRNA) and antigen in clinical samples, followed by a convalescent phase marked by the presence of antibodies in both saliva and serum. Therefore, concurrently analyzing these varied biomarkers in clinical samples throughout the disease’s course offers more precise insights for disease monitoring and management. This holistic approach would enhance our understanding of infection, infectivity stages, and the host immune response, ultimately aiding in more accurate diagnostic and therapeutic decision-making^1^.

**Figure 1.**
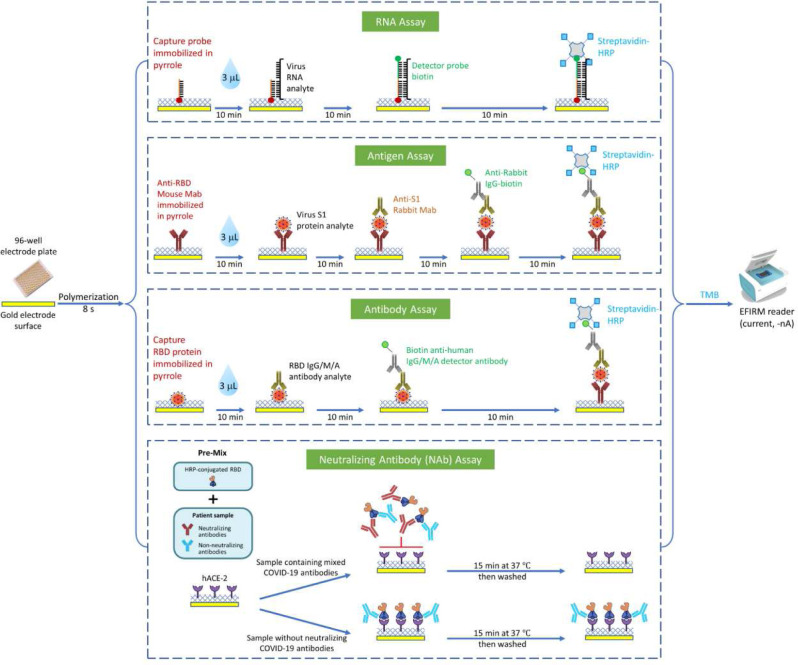
Schema and biorecognition elements of saliva SARS-CoV-2 viral RNA, N antigen, binding antibody, and neutralizing antibody assay

Saliva is a conveniently accessible bio sample that has been explored for diagnostics of COVID-19 and other diseases. Electric Field Induced Released and Measurement (EFIRM) platform is an electrochemical, plate-based, liquid biopsy platform (Figure 1) which we have optimized for direct detection of SARS-CoV-2 biomarkers in saliva. This platform can detect multiple viral and host targets without sample processing and yields performance that meets or exceeds current Emergency Use Authorization (EUA) COVID-19 diagnostic tests.

Nasopharyngeal swabbing, followed by reverse transcription of the extracted RNA and quantitative PCR (RT-qPCR), is the gold standard for detection of SARS-CoV-2 infection. However, this approach poses various challenges, such as the requirement for skilled medical professionals and a vast supply of protective equipment. Additionally, the method causes discomfort for patients and exposes healthcare staff to a high risk of infection. Saliva as a simpler and less invasive alternative has been used successfully as a diagnostic tool for SARS-CoV-2 and other various viral infections^2–4^. Notably, one study has demonstrated that the SARS-CoV-2 virus can be detected earlier in saliva samples^5^.

Loop-mediated Isothermal Amplification (LAMP) is a rapid, cost-effective, and sensitive RNA detection method that has gained attention during the COVID-19 pandemic. Unlike RT-PCR, LAMP amplifies viral RNA at a constant temperature, eliminating the need for sophisticated thermal cyclers. LAMP assays can be performed in a shorter timeframe and with minimal equipment, making them suitable for point-of-care testing and resource-limited settings. However, the analytical sensitivity of Reverse Transcription Loop-Mediated Isothermal Amplification (RT-LAMP) assay with SARS-CoV-2 RNA is around 50 copies/reaction which is below that of the standard RT-qPCR tests^6^. Building upon the advantages of LAMP assays in terms of simplicity, rapidity, and suitability for resource-limited settings, we optimized and enhanced the analytical sensitivity of the RT-LAMP assay and developed a highly sensitive and highly specific assay with multiplex and point-of-care potential for SARS-CoV-2 direct detection using self-collected whole saliva specimen. By addressing this limitation, we aim to bridge the sensitivity gap between RT-LAMP and standard RT-qPCR tests, ultimately enabling the reliable and accurate detection of low viral loads.

COVID-19 antigen assay is a diagnostic test that detects the presence of specific viral proteins in a person’s respiratory or nasal secretions. It is a rapid test that can provide results within minutes, making it a useful tool for screening and diagnosing COVID-19 infections. The antigen test uses a swab specimen taken from the nasal passages, and the results are based on the reaction between the antigen in the test kit. One limitation of current COVID-19 antigen assays is that the sensitivity and specificity of the test can vary depending on the quality and timing of the sample collection, the type of swab used, and the viral load in the patient’s body. False negatives may occur with asymptomatic or lower viral load infections. As a result, it is suggested that a negative test result should be validated through a more sensitive and specific molecular test such as PCR. Additionally, antigen testing has not been validated for screening asymptomatic individuals. We developed a highly sensitive and specific saliva-based nucleocapsid (N) antigen assay with an improved LOD. The successful development of such an assay would make a significant contribution to the field of diagnostics, providing a noninvasive and efficient method to detect individuals with lower viral loads who might otherwise be overlooked by existing diagnostic approaches.

The detection of specific antibodies following SARS-CoV-2 infection enables various applications such as evaluating the seroprevalence, identifying potential convalescent plasma donors, monitoring herd immunity, generating risk prediction models, and playing a crucial role in global vaccination strategies^7^. Previously, we have introduced the innovative, quantitative, diagnostic EFIRM platform for anti-SARS-CoV-2 Spike IgG that tracked vaccinated patients to assess the kinetics of anti-SARS-CoV-2 antibodies following inoculation. This platform utilizes a unique cyclic electric field to enhance sensitivity and specificity of saliva antibody detection, which overcame the low sensitivities and specificities of multiple serological tests with ELISA and lateral flow methods^8–12^. To push the limit of sensitivity and specificity further, we have expanded the antibody assays to detect IgG, IgM, and IgA to increase the range of time frame of detectable antibodies as IgA appearing slightly earlier than IgG and IgM. Recent findings suggest mucosal IgA to SARS-CoV-2 dominates early neutralizing activities^11^. Mucosal IgA is the major immunoglobulin in saliva, elicited by mucosal epithelial and salivary glands^12^. Thus, the saliva-based EFIRM anti-RBD assay was developed to detect IgA in addition to IgG and IgM targets.

Among host antibodies against SARS-CoV-2, anti-SARS-CoV-2 neutralizing antibodies (NAbs) are particularly significant because they inhibit the binding of the receptor-binding domain (RBD) of the surface spike (S) protein to the human angiotensin-converting enzyme 2 (hACE2) receptor. The complex formed between the virus S protein and hACE2 is responsible for the virus entry into host cells, and inhibiting the formation of this complex may prevent infection and reduce disease severity^7^. Standard SARS-CoV-2 serology assays, which primarily detect binding antibodies (BAbs) like IgG and total antibody, are unable to distinguish between general binding antibodies and neutralizing antibodies^13^. Therefore, neutralizing antibody (NAb) assays are the only reliable method for assessing the true protective immunity of antibodies^14^.

The current gold standard for measuring NAb is the conventional virus neutralization test known as Plaque Reducing Neutralization Test (PRNT), which requires a live pathogen and a biosafety level 3 (BSL3) laboratory. cPass SARS-CoV-2 Neutralization Antibody Detection Kit was developed as a surrogate virus neutralization test that can detect total NAbs in plasma in 1–2 hours in a BSL2 laboratory without the use of any live virus or cells. The cPass Neutralization Antibody Detection Kit results have shown 95.7% positive percent agreement (PPA) and 97.8% negative percent agreement (NPA) with the gold standard PRNT in clinical study. However, PRNT and cPass assays exclusively detect NAbs in plasma and serum and there is no test for measuring NAbs in saliva. Due to the lower antibody levels in saliva compared to plasma, the measurement of antibodies in saliva necessitates a more sensitive assay^13,15–17^. We developed the EFIRM NAb assay that can detect NAbs in saliva samples by successfully replicating the virus-host interaction within an EFIRM plate well. The development of a highly sensitive and specific non-invasive saliva based NAb assay would be of great value for large-scale applications, such as predicting the efficacy of vaccines and estimating the requirement for booster doses.

EUA approved molecular tests for SARS-CoV-2 are single plex platforms, conveying a single dimension of SARS-CoV-2 infection in an individual. The high precision and sensitivity of EFIRM platform enabled us to develop a novel, cost-effective, and highly sensitive and specific diagnostic assay with the capability to simultaneously detect 4-dimensions of SARS-CoV-2 including RNA, antigen, BAbs, and NAbs against the virus directly from saliva samples.

The successful development of such assay would make a significant contribution to the field of diagnostics by detecting infected individuals with lower viral loads and assessing individuals’ immunization status. This versatile platform lays the foundation for tackling potential future pandemics, thanks to its ability to easily develop EFIRM assays for any emerging infectious diseases.

## Materials and Methods

### STUDY COHORTS

#### Pre-pandemic ADA saliva samples

Saliva was collected from healthy individual volunteers at meetings of the American Dental Association (ADA) between 2006 and 2011. The study protocol was approved by UCLA IRB #06–05-042 and all methods were performed in accordance with relevant guidelines/regulations. All subjects consented prior to sample collection and saliva samples were collected as previously described^18^.

#### Pre-pandemic SMC saliva samples

Saliva was collected from patients admitted to Samsung Medical Center in Korea from 2014 to 2018. Prior to sample collection, all participants provided written informed consent. The study received IRB approval from both UCLA and Samsung Medical Center (UCLA IRB# #06–07-018–11, SMC IRB# 2008–01-028–016) and all experiments were performed in accordance with relevant guidelines and regulations. About 1 mL of whole saliva was expelled into a 50cc conical tube placed on ice. Processing occurred within 30 minutes, involving centrifugation at 2,600 ×g for 15 minutes at 4°C. The resulting supernatant was transferred to a 2 mL cryotube. 1 μL of Superase-In (Ambion) was added to the samples, followed by gentle inversion for thorough mixing. The cryotube was then frozen with dry ice and stored at −80°C.

#### Pre-pandemic plasma samples

Plasma samples obtained from healthy individuals before 2019 were acquired from innovative research. Donors contributed whole blood samples collected in K2EDTA tubes. Following the vendor’s instructions, the whole blood underwent centrifugation at 5,000 ×g for 15 minutes, and the resulting plasma was separated using a plasma extractor^19^.

#### Hospitalized COVID-19 patient samples

Archived saliva samples were sourced from an ongoing observational study involving hospitalized COVID-19 patients at UCLA. Participants were recruited within 72 hours of admission to UCLA Health hospital, and their biospecimens were collected during hospitalization and outpatient follow-ups for up to one year. The repository comprised blood (plasma and PBMC), saliva, and nasopharyngeal swabs. All participants provided informed consent via a UCLA IRB-approved protocol (IRB#20–000473) and the study was performed in accordance with the relevant guidelines. All saliva samples used in this study were collected from hospitalized patients within 3 to 15 days after symptom onset with positive RT-qPCR nasopharyngeal swab.

#### Vaccinated recovered COVID-19 outpatient samples

Saliva samples from recovered mild COVID-19 patients were acquired as part of an ongoing observational study of outpatient COVID-19. Individuals who had experienced mild COVID-19 without requiring supportive care were recruited for the study. During study visits, participants contributed blood samples (for serum, plasma, and PBMC) and saliva to a specimen repository. Informed consent was obtained from all participants. The study received IRB approval from UCLA (IRB#20–000500) and all experiments were conducted following the appropriate regulations. While enrolled in the study, participants received vaccinations, and post-vaccination samples were collected. All saliva and plasma samples from the vaccinated recovered COVID-19 outpatient cohort used in this study were obtained from individuals with positive RT-qPCR nasopharyngeal swabs who received one or two vaccinations.

#### Vaccinated infection naïve patient samples

Archived saliva samples from infection naïve vaccinated persons were obtained from an ongoing observational study at UCLA. Healthy individuals, with no history of SARS-CoV-2 infection, who were undergoing SARS-CoV-2 vaccination (any vaccine) were recruited before receiving their initial vaccine dose. They were then followed up after each vaccination and beyond. During study visits, participants contributed blood and saliva to a specimen repository. Informed consent was obtained from all participants. All procedures were performed after obtaining approval from UCLA IRB (IRB#20–000500) and were conducted in compliance with applicable guidelines and regulations^19^.

### EFIRM PLATFORM

EFIRM is an innovative platform capable of quantifying target molecules in both blood and saliva samples. The technology involves immobilizing capture moieties on an electrode structure, enabling the capture of target analytes. Quantification of the target analyte is accomplished through electrochemical measurements of the oxidation-reduction reaction between hydrogen peroxide and a tetramethylbenzidine substrate, along with the involvement of a peroxidase enzyme in a completed assay sandwich. This assay is performed on electrodes packaged in a traditional 96-well microtiter plate format (EZLife Bio, Woodland Hills, CA)^18,19^. The schematic of the EFIRM SARS-CoV-2 vRNA, antigen, BAb, and NAb assays is shown in Figure 1.

### DESIGN OF EFIRM SARS-COV-2 ASSAYS

#### Design of EFIRM vRNA assay

In order to enhance the sensitivity of the RT-LAMP assay, we designed multiple amplification targets within highly conserved regions and assessed the performance of various combinations of LAMP targets. The most favorable results were obtained when targeting two genomic regions within the N gene of SARS-CoV-2, namely N2 and NL. These regions were identified to confer highest specificity to SARS-CoV-2 detection. The N2 and NL RT-LAMP targeting sequences are highly conserved among different SARS-CoV-2 variants. An in-silico inclusivity analysis was performed aligning the assay primers to 20,329 SARS-CoV-2 sequences from GISAID’s EpiCov database, including all defined variants. Analysis demonstrated only one out of six primers to include one mismatch to each targeted sequence. Among 20K variant sequences, 99.97% and 99.92% of the mismatches are not located in the last 3 nucleotides near the 3’ end. This analysis suggested that N2 and NL primer designs not only have the capability to detect SARS-CoV-2 but also its variants. While one primer set of N2 or NL alone only reaches 99.18% and 98.81% variant matches, respectively, the dual combination of N2 and NL primer sets achieved 100% match to all of the tested SARS-CoV-2 variant strains. Therefore, this LAMP-based assay has the capability to maintain high level detection even with the continued rise in variants. Furthermore, RT-LAMP of N2 and NL led to amplicons that can be cleaved by two sets of restriction enzymes to yield 60-bp (HaeII and HincII) and 48-bp (Pst I and BcoD I) short DNA fragments that are optimal lengths for EFIRM detection^20^.

The virus in saliva samples from patients were inactivated by incubation for 15 minutes at 92 °C. The NL primer set for RT-LAMP targeting the last part of the N gene of SARS-CoV-2 sequence (GenBank accession number MN908947) was designed with PrimerExplorer V5 (http://primerexplorer.jp/e/). The N2 primer set was designed as described^6^. 20 μL of saliva samples were mixed with the same volume of TAE buffer and were pretreated by heating at 97 °C for 10 minutes and subsequently adding 4 μL of 10% Tween-20. The RT-LAMP reactions were conducted as described by the manufacturer’s protocols with WarmStart Colorimetric LAMP 2X Master Mix with UDG (NEB, Massachusetts, USA). 20 μL reactions contained 10 μL LAMP master mix, 1 μL of 20X primer mix [4 μM F3 and B3, 32 μM Forward Inner Primer (FIP) and Backward Inner Primer (BIP), and 8 μM of Loop Forward (LF) and Loop Backward (LB) primers)], 1μL 0.8M Guanidine hydrochloride (Sigma), 5 μL nuclease-free water, and 3 μL pretreated saliva samples. The RT-LAMP reactions were incubated at 65 °C using thermocycler for 40 minutes. The positive control was heat-inactivated SARS-CoV-2 virus (SARS-CoV-2 USAWA1/2020, BEI Resources, cat# NR-52286) spiked into pooled saliva collected from donors who tested negative for SARS-CoV-2. The restriction enzyme digestion was performed with four endonucleases (Hae II, Hinc II, BcoD I, Pst I) from New England Biolab. 30 μL reactions contained 3 μL of 10 × Cutsmart Buffer, 0.5 μL Hae II, 0.5 μL Hinc II, 0.5 μL Pst I, 1 μL BcoD I, 19.5 μL water and 5 μL products from RT-LAMP reaction. The mixture was incubated at 37 °C for 15 minutes. The amplified and digested N2 and NL targets were determined by EFIRM assays as described^21^. The sequences of capture and detect probes are listed in Supplementary Table 1.

EFIRM vRNA assay was developed and tested on RT-qPCR-positive archived saliva samples collected from acutely infected hospitalized COVID-19 patients within 3 to 15 days after symptom onset (n = 10) vs. infection-naïve patient samples (n = 33).

#### Design of EFIRM nucleocapsid antigen assay

Diluted saliva (1:10) in casein PBS was pipetted into a 96-well electrode microtiter plate containing pre-immobilized anti-SARS-Cov-2 antibody mouse monoclonal antibody (mAb) (SinoBiological, Beijing, China) in pyrrole (W338605; Sigma-Aldrich Corp., St. Louis, MO). It was incubated for 10 minutes and then rinsed using PBS-T wash buffer — 1x phosphate-buffered saline (Affymetrix Inc, Sunnyvale, CA) and 0.05% Tween 20 (Bio-Rad, Hercules, CA). 30 μL of 1:500 diluted anti-SARS-CoV-2 antibody Rabbit mAb (SinoBiological, Beijing, China) was pipetted into each microplate well. After a 10-minute incubation, the wells were rinsed using PBS-T wash buffer. 30 μL of diluted biotinylated Goat-anti-Rabbit mAb (Abcam, Waltham, MA) was pipetted into each microplate well. Incubation for 10 minutes followed, and then the wells were rinsed using PBS-T wash buffer. Subsequently, 30 μL of diluted streptavidin-Poly80 Horseradish peroxidase (HRP) solution was pipetted into each microplate well. Another 10-minute incubation was performed, and the wells were rinsed using PBS-T wash buffer. Finally, 60 μL of 3,3′,5,5′-tetramethyl-benzidine (TMB)/H2O2 (Thermo Fisher Scientific, Waltham, MA) readout substrate was added, and electrochemical measurement of the plate was carried out at −200 mV for 1 minute.

EFIRM antigen test was developed using saliva samples from acutely infected hospitalized COVID-19 patients (n = 10) and infection-naïve patients (n = 33).

#### Design of EFIRM BAb assay

The EFIRM BAb assay is similar to the methods in our previous publications^18,21–28^. The EFIRM anti-RBD IgG/IgM/IgA antibody analytical assays were developed using recombinant monoclonal human IgG, IgA, or IgM antibody against Spike SARS-CoV-2 RBD (CR3022) (InvivoGen, San Diego, CA). Diluted detector antibody, IgG Fc goat anti-human biotin (1:500, eBiosciencesTM, San Diego, CA), rabbit anti-human IgA monoclonal biotin (1:800, RevmAb Biosciences, San Francisco, CA), or goat anti-human IgM (1:500, Thermo Fisher Scientific, Waltham, MA) in Casein/PBS (Thermo-Fisher, Waltham, MA) was pipetted into each well and incubated for 10 minutes at room temperature to determine the analytical linearity range, limit of detection, and the standard curve. All positive samples were repeated to minimize false positives due to analytic variability.

BAb assay was developed and tested on archived saliva samples collected from acutely infected hospitalized COVID-19 patients (n = 35), vaccinated recovered COVID-19 outpatients (n = 13), and vaccinated infection naïve patients (n = 13) along with pre-pandemic ADA saliva samples (n = 88) as the control cohort.

#### Design of EFIRM NAb assay

Our test was designed to mimic the virus-host interaction in an EFIRM plate well by using purified RBD from SARS-CoV-2 S protein and the host cell receptor ACE2. The EFIRM NAb assay development involved immobilizing hACE2 protein onto a gold electrode. A mixture of hACE2 protein (GenScript, Piscataway, NJ) was diluted in a 1 mL master mix containing 5 μl of pyrrole, 50 μl of 3M potassium chloride, and 945 μl of UltraPure water (Thermo Fisher Scientific, Waltham, MA). The hACE2 mixture was added to the wells, ensuring that each well contained 500 ng of hACE2. For receptor immobilization, a cyclic square-wave electrode field was applied for 5 cycles of 1 second at 350 mV and 1 second at 950 mV (10 seconds total). After electrochemical polymerization, each electrode underwent a 6-cycle wash in PBS-T buffer. Saliva samples underwent centrifugation at 2,600 ×g for 15 minutes at 4°C. The resulting supernatant, containing cell-free saliva, was used for further analysis. Saliva samples were diluted at 1:2, plasma samples at 1:10, and cPass positive and negative controls at 1:10 using a sample dilution buffer (GenScript, Piscataway, NJ). HRP conjugated wild-type RBD was diluted 1:800 with RBD dilution buffer (GenScript, Piscataway, NJ). 60 μL of diluted saliva, plasma, and positive and negative controls, were pre-incubated with 60 μL of diluted RBD-HRP for 30 minutes to allow the interaction and binding of neutralization antibodies to RBD-HRP. Subsequently, 100 μL of the mixture was added to the EFIRM capture plate pre-coated with hACE2 protein. All samples and controls were tested in duplicates. If the sample contained SARS-CoV-2 neutralizing antibodies, they would bind to the RBD-HRP during the initial 30 minutes, inhibiting the interaction with hACE2. However, if the sample lacked neutralizing antibodies, the RBD-HRP would bind to the ACE2-coated wells during a 15-minute incubation at 37°C. Wash step was repeated. Finally, 100 μL of the TMB solution was applied, and after 5 minutes, a current readout was performed on the reader with a potential of −200 mV for 60 seconds (Figure 1).

The percent signal inhibition for the detection of neutralizing antibodies was calculated from the formula below.

%Inhibition=(1–electriccurrentofsample/electriccurrentofnegativecontrol)×100.

The test was calibrated for the quantitative detection of anti-SARS-CoV-2 neutralizing antibodies using the SARS-CoV-2 Neutralizing Antibody Calibrator (GenScript, Piscataway, NJ). The NAb concentrations were as follows: 300U/mL, 150U/mL, 75U/mL, 37.5U/mL, 18.75U/mL, 9.375U/mL, and 4.688U/mL. The data generated from the NAb calibration curve was plotted with EFIRM current on the Y-Axis versus concentration on the X-Axis using a 4PL model with GraphPad Prism. Quantitative results were expressed in Units/mL^19^.

Saliva NAb assay was developed using saliva samples collected from vaccinated recovered COVID-19 outpatients and vaccinated infection naïve patients (n = 31) along with pre-pandemic SMC saliva samples (n = 60) as the control group. Plasma NAb assay was developed and tested on paired plasma samples obtained at the same visit from vaccinated recovered COVID-19 outpatients and vaccinated infection naïve patients (n = 30) and plasma samples from pre-pandemic plasma cohort (n = 60).

### STATISTICAL ANALYSIS

All the signal readout was calibrated with a SARS-CoV-2 antigen standard (SARS-Related Coronavirus 2, Isolate USA-WA1/2020, Gamma-Irradiated, NR-52287, BEI resource), recombinant monoclonal human IgG, IgA, and IgM antibody against Spike RBD (CR3022) (InvivoGen, San Diego, CA), or Neutralizing Antibody Calibrator (GenScript, Piscataway, NJ). Test results were only performed after the positive (SARS-CoV-2 standard) and negative controls (non-SARS-CoV-2 standard) and standard curve had been examined and determined to be valid and acceptable. If the controls were not valid, the patient results could not be interpreted, and the entire assay was repeated. The level of analytes between the groups were compared using the two-tailed test. P values < 0.05 were considered significant. The discriminatory performance of measured analytes in saliva was assessed using the area under the receiver operating characteristic (ROC) curves^29^ with the associated 95% confidence interval by the Wilson/Brown method on GraphPad Prism 8^30^.

## Results

### EFIRM SARS-COV-2 VRNA ASSAY

#### Development of EFIRM vRNA assay

The Saliva SARS-CoV-2 infection/vRNA assay allows direct detection of SARS-CoV-2 vRNA in 3 uL of whole saliva in a tandem reaction of RT-LAMP, restriction enzyme digestion and EFIRM. Two genomic regions of the nucleocapsid gene of SARS-CoV-2 RNA, N2 and NL, were identified to confer highest specificity to SARS-CoV-2 detection. RT-LAMP of N2 and NL led to amplicons that can be cleaved by two sets of restriction enzymes to yield 60-bp (HaeII and HincII) and 48-bp (Pst I and BcoD I) short DNA fragments which are optimal lengths for EFIRM detection.

#### Determination of analytical performance

**Figure 2.**
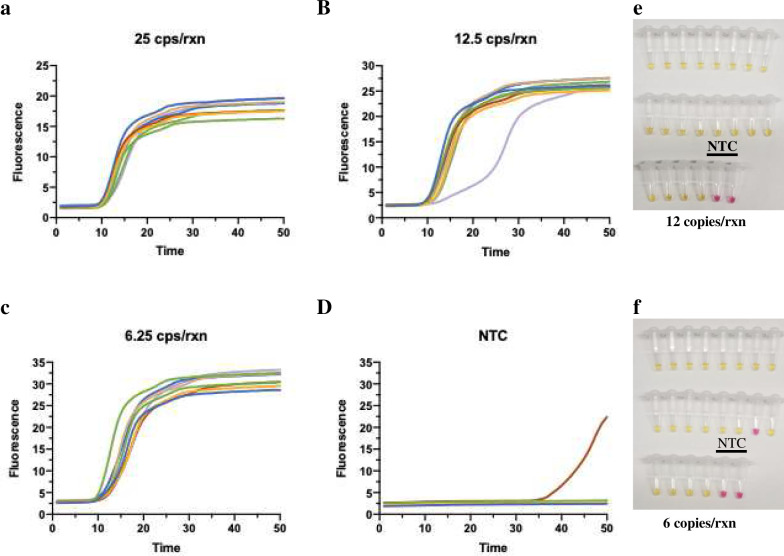
The analytical performance of RT-LAMP vRNA assay with extracted viral RNA. The N2 + NL RT-LAMP assay performance using quantitative PCR (qPCR) control SARS-CoV-2 viral RNA from BEI resources (cat# NR-52346) at A, 25 copies/reaction, B, 12.5 copies/reaction, C, 6.25 copies/reaction, and D, no-template negative control. The assays were conducted with SYTO-9 dye for monitoring the reaction on qPCR machine. 12 replicate reactions were performed at each concentration. The LOD of the assay was further determined with colorimetric RT-LAMP reaction on 20 replicates with 12 (E) and 6 (F) copies/reaction of SARS-CoV-2 RNA.

To evaluate the analytic performance of the RT-LAMP assay with N2 and NL, we conducted the assay with different concentrations of purified SARS-CoV2 RNA standards (Figure 2a–d). SYTO-9 double-stranded DNA binding dye was used for monitoring the reaction in real-time on a qPCR machine. As shown in Figure 2c, all 12 replicates of LAMP assay with as low as 6.25 copies/reaction were successfully amplified in 25 min. The other advantage of the LAMP assay was that it could detect the colorimetric change of the reaction^6^. The LOD of the RT-LAMP assay was further determined by 20 replicates with 12 and 6 copies/reaction of RNA template by colorimetric reaction (Figure 2e–f). The LOD of the assay reached 6 copies/reaction (detect 19 out 20 replicates) which was at the same level of all quantitative PCR-based assays and 8 times better than published sensitivity of the RT-LAMP assay from New England Biolabs^6^.

**Figure 3.**
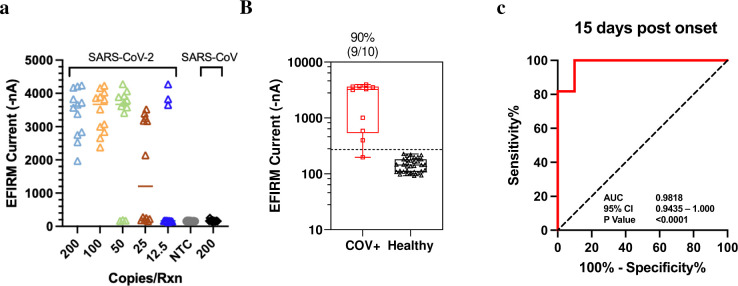
Analytical and Clinical performance of LAMP-EFIRM direct Saliva SARS-CoV-2 vRNA assay. A, The LOD was determined with saliva spiked with heat inactivated SARS-CoV-2 virus. NTC, no-template control. B, Viral RNA test analysis results for RT-qPCR-positive samples of acutely infected hospitalized patients (n = 10) vs vaccinated infection-naïve patient samples (n = 33). Box plot of vRNA test results corresponding to EFIRM measurement. The dotted line indicates cutoff of mean + 3 × SD. C, ROC analysis of vRNA assay performance within 15 days post onset of symptoms resulted in an AUC of 0.9818.

We further tested the assay for viral direct detection with saliva specimens. The heterogeneity of saliva from different donors can produce different colors between yellow and pink in the colorimetric LAMP assay (data not shown) leading to ambiguous results. To reduce the rate of false positive and false negative results from direct RT-LAMP assay, EFIRM assay was developed by targeting the 60-bp and 48-bp short DNA fragments from restriction enzyme digestion of N1 and NL target, respectively. The analytic performance of this LAMP-EFIRM direct saliva vRNA assay is shown in Figure 3. The LOD of the assay with 100 copies/reaction (12 positive out 12 replicates) was determined using saliva spiked with heat inactivated SARS-CoV-2 virus (Figure 3a).

#### Clinical validation of vRNA test with saliva

We conducted further testing of the direct detection assay using clinical samples. A total of 43 samples were tested, including 10 from hospitalized COVID-19 patients within 3 to 15 days after symptom onset with confirmed RT-qPCR positive nasopharyngeal swabs, and 33 samples from infection naïve participants. Out of the 10 saliva samples obtained from hospitalized patients, 90% (9/10) showed LAMP-EFIRM positivity (Figure 3b). The vRNA assay distinguished COVID-19 positive patients (n = 10) from healthy (n = 33) with an area under the ROC curve (AUC) of 0.9818 (95% CI: 0.9435–1.000) (Figure 3c).

### EFIRM SARS-COV-2 ANTIGEN ASSAY

#### Development of SARS-CoV-2 EFIRM antigen assay

The Saliva SARS-CoV-2 N Antigen assay detects the N protein by antibody sandwich assay using anti-N mouse mAb to capture SARS-CoV-2 N protein followed by detector antibodies, rabbit anti-N mAb and biotinylated goat anti-rabbit IgG.

#### Determination of analytical performance

**Figure 4.**
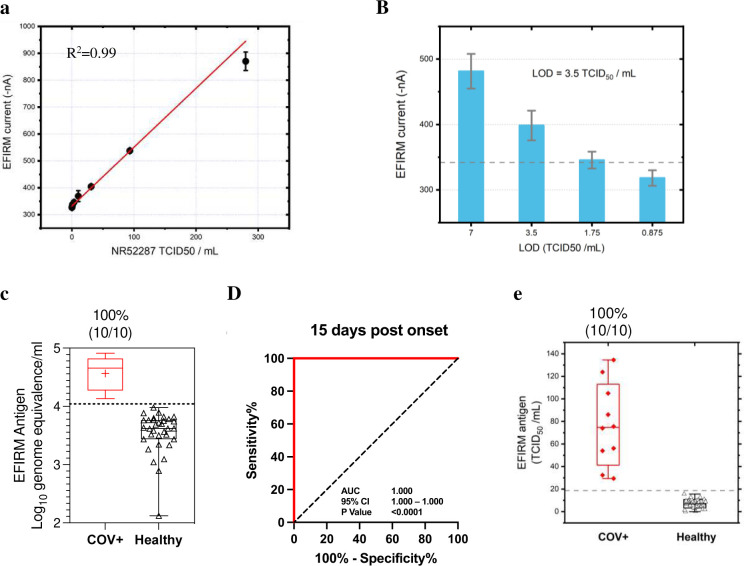
Analytical and clinical performance of EFIRM direct Saliva SARS-CoV-2 N Antigen assay. A, Analytical linearity with NR-52287 (gamma inactivated virus) from 0–300 TCID^50^/mL. B, LOD determined by 24 replicates at LOD, 2 LOD and ½ LOD. C, Antigen test analysis results for RT-qPCR-positive samples of acutely infected hospitalized patients (n = 10) vs vaccinated infection naïve patient samples (n = 33). Box plot of antigen test results corresponding to Log10 genome equivalence. The dotted line indicates cutoff of mean + 3 × SD. D, ROC analysis of antigen assay performance within 15 days post onset of symptoms resulted in an AUC of 1.000. E, Box plot of antigen test corresponding to EFIRM antigen level (TCID50/mL).

The linearity of the assay is displayed in Figure 4a with the range from 300 to 0 TCID₅₀/mL. The assay confers exquisite LOD of 3.5 TCID₅₀/mL (Figure 4b), which is 7 times more sensitive than the highest performance EUA test at LOD of 22.5 TCID₅₀/mL (nasal swab)^31–37^ (Supplementary Table 2). Testing was conducted with heat inactivated SARS-CoV-2 strain isolated from positive nasopharyngeal swab specimen with titer of 2.8 × 10^5^ TCID₅₀/mL or 1.7 × 10^9^ genome equivalents/mL (BEI resources, cat# NR-52287).

#### Clinical validation of antigen test with saliva

Saliva clinical samples from acute hospitalized COVID-19 patients within 3 to 15 days after symptom onset with RT-qPCR positive nasopharyngeal swabs, exhibited positive detection of N antigen in all samples (n = 10) with negative detection from healthy control individuals (n = 33) (Figure 4c). Saliva collected from vaccinated infection naïve outpatient samples (n = 33) were used to determine the analytical specificity of 100% with cutoff positivity at 3 standard deviations above the mean. Samples above the cutoff level of 4.04 log10 genome equivalents/mL are considered as true positives. The antigen test has a clinical performance with an AUC of 1.000 (95% CI: 1.000–1.000) (Figure 4d). The mean ± SD of N antigen level in acute hospitalized patients was 77.05 ± 35.90 TCID₅₀/mL compared to 7.02 ± 3.76 TCID₅₀/mL in healthy controls (p < 0.0001) (Figure 4e). Some have suggested that antigen positivity could be a method to identify persons with active infection who are most at risk to transmit to others^38^, as PCR-based tests are known to remain positive beyond the infectious window. The antigen test serves to concordantly affirm the SARS-CoV-2 vRNA results and provides additional information regarding active versus recent infection.

### EFIRM SARS-COV-2 BINDING ANTIBODY ASSAY

#### Development of SARS-CoV-2 EFIRM BAb assay

The EFIRM anti-SARS-CoV-2 RBD IgG/IgM/IgA antibody assays were developed using recombinant SARS-CoV-2 RBD immobilized onto the gold electrode. Biotinylated anti-human detector antibodies were used to detect anti-SARS-CoV-2 RBD IgG, IgM or IgA in saliva samples. The signal was then enhanced through a standard streptavidin/horseradish peroxidase reaction that generates an electric current measured by the EFIRM reader at the nanoampere (nA) scale.

#### Determination of analytical performance

##### Linearity

**Figure 5.**
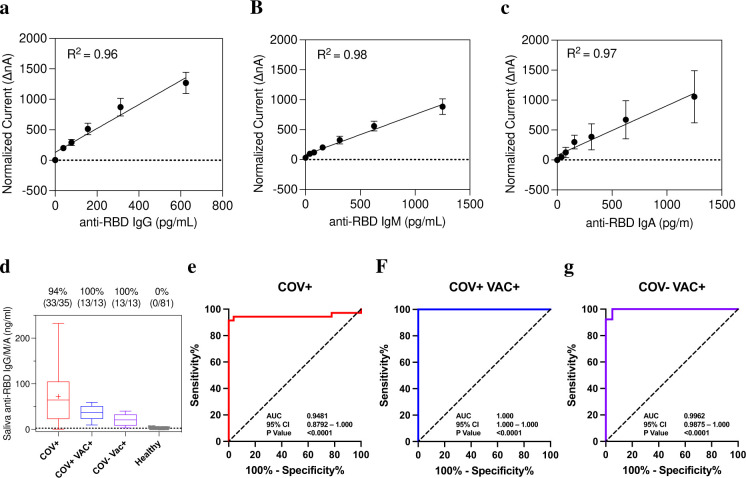
Analytical and Clinical performance of EFIRM direct saliva SARS-CoV-2 antibody assay. A-C, Linear range for anti-RBD IgG, B, IgM, and C, IgA assays. D, Antibody test results for ELISA serum-positive samples of acutely infected hospitalized patients (n = 35, COV+), vaccinated recovered COVID-19 outpatients (n = 13, COV+ VAC+), and vaccinated infection naïve patient samples (n = 13, COV− VAC+) vs healthy control samples (n = 81). Box plot of antibody test results corresponding to measured IgG/IgM/IgA in ng/mL. E–G, ROC analysis of antibody test performance resulted in AUC of 0.9481, 1.000, and 0.9962 for COV+, COV+ VAC+, and COV− VAC+ groups, respectively.

Figure 5a–c demonstrates analytical linearity range of anti-RBD IgG, IgM, and IgA and limit of detection of 39 pg/mL. The Y-axis shows amperage measured in nA and the X-axis is spiked-in concentration of IgG in pg/mL. This allows us to create a standard curve containing the following points: 5 ng/mL, 2.5 ng/mL, 1.25 ng/mL, 0.625 ng/mL, 0.3125 ng/mL, 0.156 ng/mL, 0.7813 ng/mL, and 0 ng/mL. Unknown clinical samples are correlated to the concentration of the antibody by comparison of the normalized current to the curve.

##### Specificity and Reference Range

**Figure 6.**
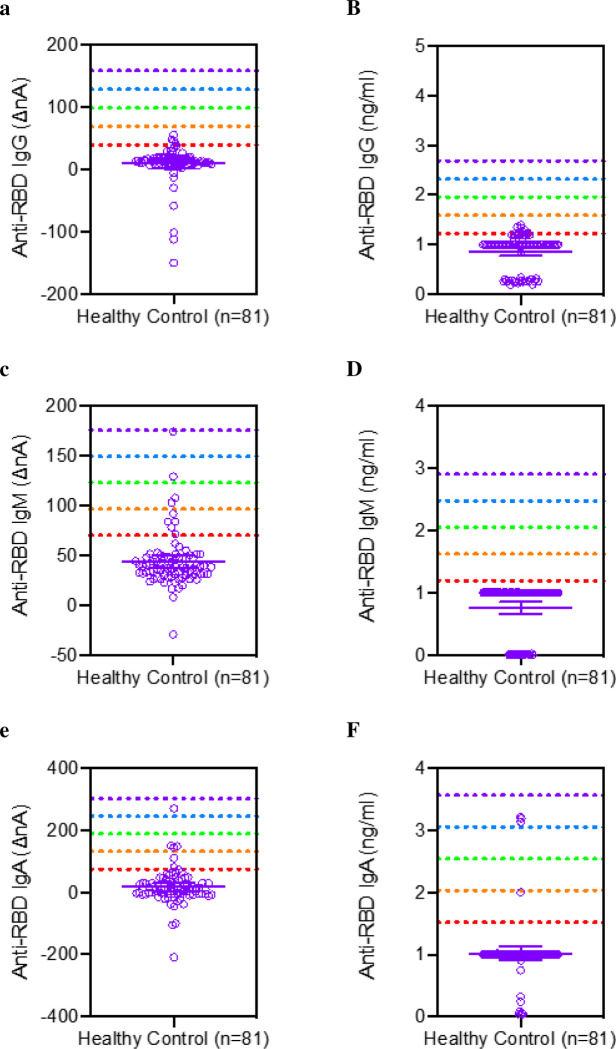
Healthy reference range of saliva anti-RBD antibody assay of 81 healthy subjects in normalized current (ΔnA) and ng/mL of A-B, IgG, C-D, IgM, and E-F, IgA assays.

We analyzed a series of 81 samples collected between 2006 and 2009 at the annual meeting of the ADA. Scatter plots of these data for both nA and ng/mL are shown in Figure 6. We established the mean and standard deviation for both raw nA values and concentration in ng/mL. The analytical specificity was determined by reference range of 5 SD above the mean. A five-sigma level is considered the gold standard significance and would lead to a specificity of 99.9994%.

#### Clinical validation of BAb test with saliva

Saliva samples collected from acutely infected hospitalized patients (n = 35, COV+), vaccinated recovered COVID-19 outpatients (n = 13, COV+ VAC+), and vaccinated infection naïve patient samples (n = 13, COV− VAC+) were assayed by EFIRM anti-RBD IgG/IgM/IgA. Pre-pandemic ADA samples were used as controls (n = 88). The first column in the box plot of Figure 5d shows that 33 out of 35 acutely infected hospitalized patients tested positive for anti-RBD antibodies with a sensitivity of 94%. Figure 5e displays combined antibody test performance of 81 healthy controls and 35 hospitalized patients with an AUC of 0.9481 (95% CI: 0.8792–1.000). The combined antibody assay can detect 100% antibody positivity in vaccinated recovered COVID-19 outpatients and vaccinated infection naïve patients (Figure 5d columns 2 and 3). The antibody assay can distinguish COV+ VAC+ and COV− VAC+ from healthy with AUC values of 1.000 (95% CI: 1.000–1.000) and 0.9962 (95% CI: 0.9875–1.000), respectively (Figure 5f–g).

### EFIRM SARS-COV-2 NEUTRALIZING ANTIBODY ASSAY

#### Development of SARS-CoV-2 EFIRM NAb test

The EFIRM NAb assay was developed using hACE2 protein immobilized onto a gold electrode. The protein-protein interaction between RBD-HRP and hACE2 is disrupted by NAbs against SARS-CoV-2 RBD, if present in a clinical sample. The current of the sample is inversely dependent on the titer of the anti-SARS-CoV-2 NAbs.

#### Determination of analytical performance

**Figure 7.**
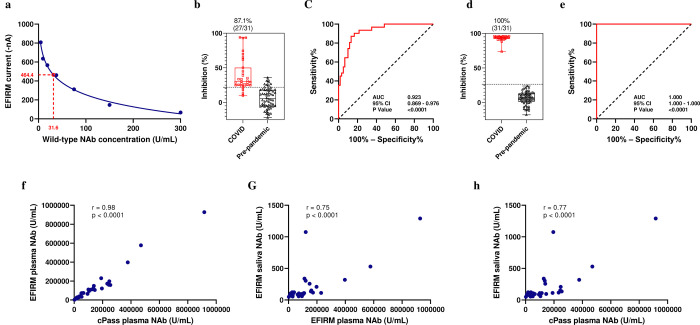
Analytical and clinical performance of EFIRM saliva and plasma SARS-CoV-2 neutralizing antibody assay. A, SARS-CoV-2 NAb Calibration Curve and calculated LOD. B, NAb test results for saliva samples of vaccinated recovered COVID-19 outpatients and vaccinated infection naïve patients (n = 31) vs pre-pandemic SMC saliva samples (n = 60). Box plot of NAb test results corresponding to measured %inhibition. C, ROC analysis of saliva NAb test performance resulted in an AUC of 0.923. D, NAb test results for plasma samples of vaccinated recovered COVID-19 outpatients and vaccinated infection naïve patients (n = 30) vs pre-pandemic plasma samples (n = 60). E, ROC analysis of plasma NAb test performance resulted in an AUC of 1.000. F, A correlation of r = 0.98 was found between NAb titers in cPass and EFIRM plasma NAb assays. G, A correlation of r = 0.75 was observed between NAb titers in paired saliva and plasma measured on EFIRM platform. H, A correlation of r = 0.77 was found between NAb titers in paired saliva and plasma measured on EFIRM and cPass platforms, respectively.

To determine the LOD, we conducted a comprehensive experiment to assess the repeatability of the assay. Two different operators independently performed two replicates of negative controls using three different cPass SARS-CoV-2 Neutralization Antibody Detection Kits on three separate EFIRM plates over the course of three days. Using the mean and standard deviation of 108 datasets, we calculated the LOD current using the formula: LOD current = mean current − 3 × SD and determined the LOD U/mL using a 4PL model in GraphPad Prism. The assay demonstrated high repeatability and reproducibility, with minimal variation due to different effectors (Supplementary Fig. 1). The calculated LOD is 31.6 U/mL (Figure 7a).

#### Comparison to current EUA test

The cPass SARS-CoV-2 Neutralization Antibody assay has an LOD of 47 U/mL for detecting NAbs^13^. In comparison, the EFIRM NAb assay exhibits superior performance with an LOD that is substantially lower than the cPass assay.

#### Clinical validation of NAb test with saliva

To validate the clinical performance of the EFIRM saliva NAb assay, we compared 31 saliva samples from vaccinated recovered COVID-19 outpatient cohort and vaccinated infection naïve patient cohort (24 vaccinated recovered COVID-19 outpatient samples and 7 vaccinated infection-naïve outpatient samples) with 60 saliva samples from the pre-pandemic SMC saliva cohort. The mean ± SD of %inhibition in the COVID group was 40.06% ± 23.65% compared to 6.42% ± 14.45% in the pre-pandemic group (p < 0.0001) (Figure 7b). Based on the %inhibition of each sample, we plotted an ROC curve and determined a cutoff value of 22% signal inhibition. The EFIRM saliva NAb assay distinguished COVID-19 recovered or vaccinated infection naïve patients from the pre-pandemic group with an AUC of 0.923 (95% CI: 0.869 to 0.976), a sensitivity of 87.10%, and a specificity of 86.67% (Figure 7c).

#### Clinical validation of NAb test with plasma

For clinical validation of the plasma NAb assay, we compared 30 paired plasma samples obtained at the same visit from COVID-19 recovered or vaccinated patients (23 vaccinated recovered COVID-19 outpatient samples and 7 vaccinated infection-naïve patient samples) with 60 plasma samples from pre-pandemic plasma cohort. The mean ± SD of %inhibition in the COVID group was 93.16% ± 4.17% compared to 6.27% ± 9.12% in the pre-pandemic group (p < 0.0001) (Figure 7d). The EFIRM plasma NAb assay differentiated COVID-19 recovered or vaccinated patients from the pre-pandemic samples with an AUC of 1.000 (95% CI: 1.000–1.000), a sensitivity of 100%, and a specificity of 100%. The cutoff value for the plasma assay was determined to be 26.5% signal inhibition (Figure 7e)

#### Clinical agreement between EFIRM plasma NAb assay and PRNT50

To validate the clinical performance of the EFIRM plasma NAb assay a clinical agreement study was conducted using as comparator the PRNT which is the gold standard for detecting NAbs. The cutoff for the PRNT comparator tests was determined as described in Supplementary Table 3. The combined cohort comprised samples from normal healthy people (n = 6) and samples from RT-PCR confirmed SARS-CoV-2 positive patients (n = 9). The EFIRM plasma NAb assay showed 100% positive percent agreement and 100% negative percent agreement with PRNT.

#### Correlation between NAb titers in cPass and EFIRM plasma NAb assays

We assessed the NAb titer in the mentioned 30 plasma samples utilizing both the EFIRM plasma NAb assay and the cPass SARS-CoV-2 Neutralization Antibody assay (GenScript, Piscataway, NJ). Results showed a strong correlation between the level of NAbs measured by the two assays (r = 0.98, p < 0.0001). Pearson correlation coefficient (r) and p-value are indicated in Figure 7f.

#### Correlation between NAb concentration in saliva and plasma

We compared the level of NAbs in the saliva and plasma samples of vaccinated recovered COVID-19 outpatient and vaccinated infection naïve patient cohorts (n = 30) through the EFRIM saliva and plasma NAb assays. A significant correlation was observed between the levels of NAbs in paired saliva and plasma, emphasizing their interrelationship (r = 0.75, p < 0.0001) (Figure 7g).

#### Saliva equivalence of neutralizing activity to SARS-CoV-2 in plasma

We also compared the level of NAbs in paired saliva and plasma samples using EFIRM and cPass platforms, respectively. A significant correlation was found between the NAb titers (r = 0.77, p < 0.0001) (Figure 7h). A recent study estimated that a neutralization level of 54 international units (IU)/mL in plasma provides 50% protection from SARS-CoV-2 infection^39^. GenScript showcased that titers interpolated from the cPass assay can be converted to WHO IU/mL by multiplying the cPass U/mL titer by a factor of 1.62613^13^. Thus, 54 WHO IU/mL will be equal to 33.2 U/mL NAbs interpolated from the cPass calibration curve. This is equivalent to 664 U/mL total NAbs in the plasma sample considering the sample dilution factor. Using a second-order local polynomial regression model (in the log scale), we conducted interpolation to ascertain the saliva equivalency of this level of total NAbs in plasma. The anticipated interpolated value for this level is 87 U/mL total NAb in saliva.

#### EFIRM saliva COVID-19 assays compared with current EUA assays

The clinical performance of EFIRM’s detection of SARS-CoV-2 compared to approved EUA assays for vRNA, antigen, binding antibodies and neutralizing immunity is shown in Table 1. 40 μL of saliva is sufficient for EFIRM to concurrently detect all 4 dimensions of SARS-CoV-2, directly, non-invasively with a performance that surpasses current EUA approved assays.

## Discussion

The EFIRM SARS-CoV-2 RNA assay test offers multiple advantages compared to currently EUA approved viral RNA tests^40^. These include direct detection in only 3 μL of saliva without the need for extraction, as well as a detection performance of 100 copies per reaction.

The EFIRM antigen assay is compared with other EUA antigen assays on analytical LOD, clinical sensitivity and specificity^41–45^. The assay has an LOD of 3.5 TCID^50^/mL, which is 7 times more sensitive than the highest performance EUA test at LOD of 22.5 TCID^50^/mL (nasal swab)^31–37^. For clinical samples, EFIRM demonstrated 100% specificity and 100% sensitivity when samples were collected within 15 days of symptom onset. In addition, EFIRM is a quantitative assay as other antigen assays are qualitative. The EFIRM antigen test is a non-invasive and easily accessible saliva-based test. It eliminates the need for sample pre-treatment, utilizing the whole saliva sample with 3 μL saliva required for each assay. Since COVID-19 antigen level is very time sensitive, the antigen assay developed here is easy for long time monitoring of the viral load.

Current EUA serology assays only include IgG and IgM analytes. EFIRM BAb assay is the only quantitative SARS-CoV-2 anti-RBD assay in saliva with comparable sensitivity and specificity to existing EUA serology assays that include IgA detection. Our goal was to create a quantitative saliva-based antibody assay with enhanced sensitivity and specificity by combining detection of IgG/M/A and a reference range of 5 sigma greater than the mean to overcome false positives. The anti-RBD antibody test is plate-based and high-throughput that performs with an AUC greater than 0.94. With healthcare workers at high risk of exposure to SARS-CoV-2 and mandatory immunization, this test can serve as an appropriate longitudinal assessment of antibody levels.

Our exclusive electrochemical saliva-based assay for quantifying SARS-CoV-2 functional neutralizing antibodies is multiplexable, quantitative, and non-invasive. It stands as the only testing method capable of accurately assessing neutralizing antibodies in saliva samples. The saliva NAb assay demonstrates sufficient sensitivity and specificity, making it valuable for population-based monitoring and individual monitoring post-vaccination. To explore the potential diagnostic utility of saliva in measuring systemic neutralizing antibodies, we investigated the correlation between NAb levels in saliva and plasma. The findings revealed a significant positive correlation in neutralizing antibody titers, suggesting that saliva could serve as a surrogate measure of systemic immunity to SARS-CoV-2. This study marked the first comparison of neutralizing antibody levels in saliva and plasma^19^.

### LIMITATIONS

This study has a few limitations that should be considered. Firstly, the sample size was relatively small, indicating the need for larger studies to confirm the reproducibility of the findings. Secondly, the cohorts used in the analysis of saliva NAb assay were from two different countries, serving as the pre-pandemic and vaccinated recovered COVID-19 outpatient and vaccinated infection naïve patient cohorts. Ideally, it would have been preferable for the cohorts to be from the same country to minimize potential confounding factors.

## Conclusion

Our comprehensive assay, capable of detecting SARS-CoV-2 vRNA, antigen, BAbs, and functional NAbs, holds immense value in diagnosing both acute and convalescent COVID-19 infections, as well as assessing an individual’s immunization status following vaccination. This versatile assay not only allows for the swift and precise identification of SARS-CoV-2 but also establishes a framework for addressing potential future pandemics. Its capability for the rapid development of EFIRM tests for various antigens makes it a valuable tool for early identification and monitoring of emerging infectious diseases. This diagnostic platform has the potential to revolutionize future pandemic preparedness and response strategies, facilitating prompt and efficient containment of novel pathogens.

## Figures and Tables

**Table 1.** Performance of EFIRM saliva SARS-CoV-2 assays compared to EUA authorized tests

Assay	LOD	Sensitivity	Specificity	Singular EUA Test (LOD or Sensitivity)	Comparison to EUA Tests	TAT	Volume	Variants	Costs per Assay	Test Setting	Multiplex

vRNA	100 copies/reaction	90% (9/10) (≤15 days post sx)	100% (33/33)	μ100 copies/reaction (SalivaDirect)	1X	60 min	3 μL	Yes	$5.30	Point-of-care Collection/Reference Lab	Yes
Antigen	3.5 TCID_5o_/mL	100% (10/10) (≤15 days post sx)	100% (33/33)	22.5 TCIDso/mL (Nasal swab)	7X	55 min	3 μL	Yes	$6.46	Point-of-care Collection/Reference Lab	Yes
Combined IgG/M/A Antibody	39 pg/mL	95% (33/35)	100% (81/81)	86–100% IgM serology; 90–100% IgG serology; No EUA IgA serology test available	1X to serology assays. No saliva EUA tests available	45 min	3 μL	Yes	$9.42	Point-of-care Collection/Reference Lab	Yes
Neutralizing antibody	31.6 U/mL	87.10% (27/31)	86.67% (52/60)	no EUA saliva neutralizing antibody test available	no EUA saliva neutralizing antibody test available	60 min	30 μL	Yes	$9.50	Point-of-care Collection/Reference Lab	Yes

## Data Availability

All data generated or analyzed during this study are included in this published article (and its Supplementary Information files).
